# Kinetics and Thermodynamics of Reserpine Adsorption onto Strong Acidic Cationic Exchange Fiber

**DOI:** 10.1371/journal.pone.0138619

**Published:** 2015-09-30

**Authors:** Zhanjing Guo, Xiongmin Liu, Hongmiao Huang

**Affiliations:** 1 College of Chemistry and Chemical Engineering, Guangxi University, Nanning, Guangxi, PR China; 2 Key laboratory of generic technology research and development of traditional Chinese medicine preparation in Guangxi colleges and universities, College of Pharmacy, Guangxi University of Chinese Medicine, Nanning, Guangxi, PR China; Virginia Commonwealth University, UNITED STATES

## Abstract

The kinetics and thermodynamics of the adsorption process of reserpine adsorbed onto the strong acidic cationic exchange fiber (SACEF) were studied by batch adsorption experiments. The adsorption capacity strongly depended on pH values, and the optimum reserpine adsorption onto the SACEF occurred at pH = 5 of reserpine solution. With the increase of temperature and initial concentration, the adsorption capacity increased. The equilibrium was attained within 20 mins. The adsorption process could be better described by the pseudo-second-order model and the Freundlich isotherm model. The calculated activation energy *Ea* was 4.35 kJ/mol. And the thermodynamic parameters were: 4.97<Δ*H*<7.44 kJ/mol, -15.29<Δ*G*<-11.87 kJ/mol and 41.97<Δ*S*<47.35 J/mol·K. The thermodynamic parameters demonstrated that the adsorption was an endothermic, spontaneous and feasible process of physisorption within the temperature range between 283 K and 323 K and the initial concentration range between 100 mg/L and 300 mg/L. All the results showed that the SACEF had a good adsorption performance for the adsorption of reserpine from alcoholic solution.

## Introduction

**Fig 1:**
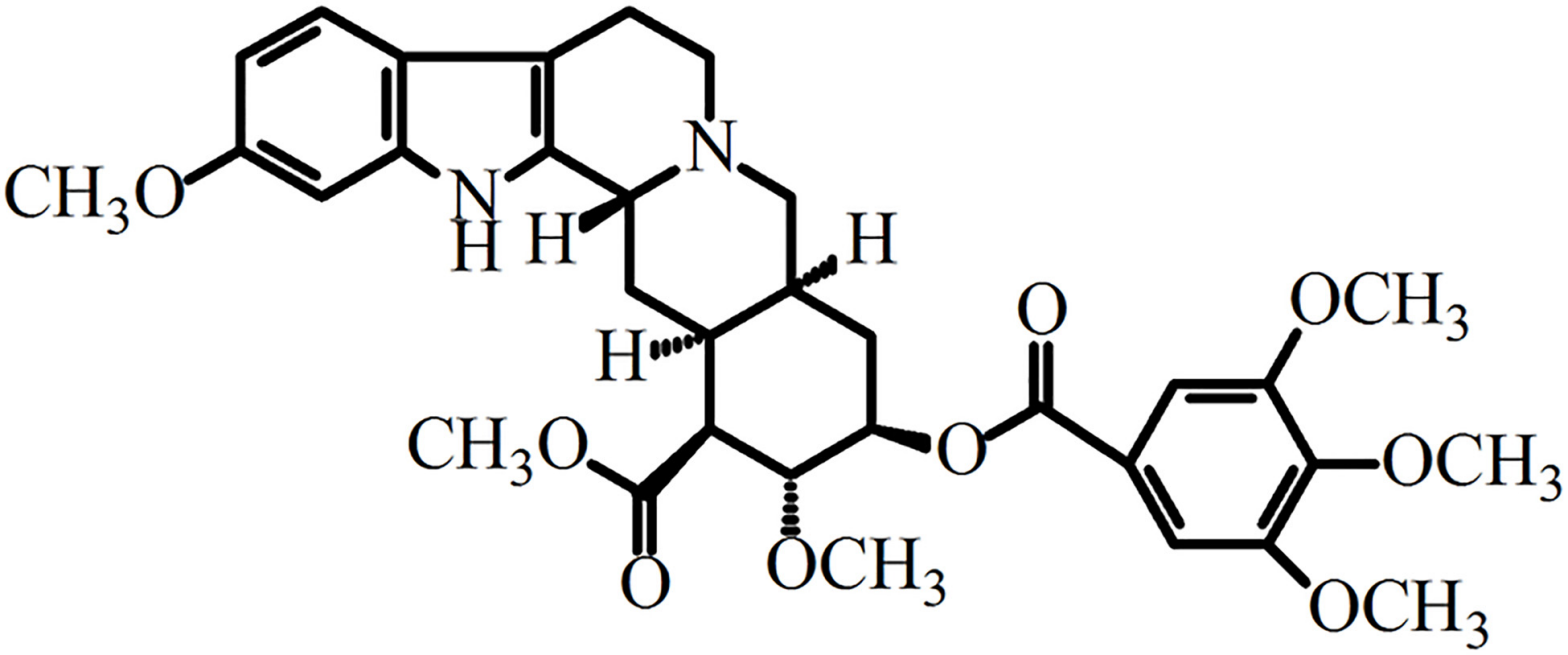
Chemical structure of reserpine.

Reserpine (structure as Fig 1), one of the most important *Rauwolfia* indole alkaloids [1], primarily acts as a sympatholytic, anti-hypertensive and sedative agent [2, 3]. Recently it was even reported to be beneficial in the cases of Huntington disease [4]. Reserpine is generally isolated from the roots of *Rauwolfia* species [5], where it is in low content (254.8–689.5 μg/g, dry wt)[6]. Reserpine shares the similar physicochemical properties of the indole alkaloids in many plants of *Rauwolfia* species. Both its low content and similar physicochemical properties make reserpine difficult to be isolated and purified from *Rauwolfia* species.

A review revealed that the most papers in English about reserpine focused on the pharmacological activities, but not on isolation and purification. So far the isolation and purification were mainly reported by papers in Chinese. The conventional methods in China for extracting and purifying reserpine from *Rauvolfia* species usually consisted of the following steps: maceration in benzene, evaporation, dissolution in methanol and HCl, filtration, adding KSCN to the filtrate, evaporation and finally dissolution in ammonia water with subsequent filtration [7]. However, the conventional methods had been weeded out because of low yields, large dosages of organic solvents, serious pollution and time-consuming procedures. Recently the macro-porous adsorption resins [8–10] and ion exchange resins [11] have been applied to separation and purification of reserpine from *Rauwolfia* species. Compared with the conventional separation methods, these new methods provide higher yields and less pollution [8–10]. However, these resins suffer from easily cracking and poor recycle rate. The resins can easily be poisoned by some organic or inorganic compounds when the pores are clogged by the colloids in the treated solution [12]. Therefore, a more efficient and more reliable method for reserpine isolation is highly desired.

Ion exchange fiber (IEF) is a new kind ion exchange and adsorption material. Compared with ion exchange resin (IER), IEF has better antipollution ability and higher adsorption/desorption rate because of the bigger specific surface area and the more locations of function groups [13], and is still suitable to further utilization after a number of saturation/regeneration cycles. Due to these favorable features, IEF has been widely applied in wastewater purification and metal recovery [14–16]. Currently, IEF has also been used to isolate active ingredients from herbal raw materials, such as baicalin [17], dihydrocapsaicin [18], drocapsaicin [19], berberine [20], salvianolic acid B [21] and catechin [22]. However, the application of IEF for separation of reserpine from *Rauvolfia* species has not been reported.

In this study, the strong acidic cationic exchange fiber (SACEF) was chosen as the adsorbent. The adsorption behavior of reserpine onto SACEF was investigated. The effects of adsorbent dosage, pH value, temperature, initial concentration and contact time on the adsorption process were studied. Kinetics and thermodynamics studies had been performed and the results had been analyzed. The thermodynamic parameters, such as Δ*H*, Δ*G* and Δ*S*, were calculated. Furthermore, the adsorption process and mechanism were analyzed by SEM and FTIR. The results proved that SACEF was an efficient adsorption material for reserpine. A theoretical basis for isolation of reserpine from *Rauwolfia* species was provided through this study.

## Materials and Methods

### 2.1 Materials

The strong acidic cation exchange fiber (SACEF, -SO_3_H ion-exchange group, exchange capacity≥4.0 mmol/g, diameter = 40±2 μm) was purchased from Guilin Zhenghan Sci. &Tech. Co., Ltd. (Guangxi, China). Reserpine (purity > 99.8%) was purchased from Aladdin company (Shanghai, China). Acetonitrile (chromatographic grade) was obtained from Merck (Germany). All the other chemicals used in this study were analytical grade, and the water used in the experiments was deionized water (the electrical conductivity < 0.1 μs/cm) made by ELGA Ultrapure Water Polishing System (model: CLXXUVFM2).

The stock solution of 2000 mg/L of reserpine was prepared by dissolving the reserpine powder in the mixture of chloroform and methanol (*V*/*V*, 1:9). The desired test solutions of reserpine were prepared using appropriate subsequent dilutions of the stock solution by 60% hydrous alcohol. The range of concentrations of desired test solutions varied between 100 mg/L and 300 mg/L. The pH of each test solution was adjusted to the required value with 0.1 mol/L NaOH and 0.1 mol/L HCl.

### 2.2 Pre-treatment of SACEF

The pre-treatment method of SACEF according to GB/T5476-2013 (method for pretreating ion exchange resins) was described below. The fiber was washed in double volumes of deionized water (70°C–80°C) until the washing liquid was colorless and then soaked with 1 mol/L HCl, deionized water, 1 mol/L NaOH, deionized water, 1 mol/L HCl for 30 minutes, respectively. At last the fiber was then washed repeatedly with deionized water until the pH of washings reached about 7 before it was dried in a vacuum oven at 60°C for the later experiments.

### 2.3 Analysis

The concentrations of reserpine in the solutions before and after equilibrium were determined by HPLC [23] (Waters 1525, USA) equipped with reversed phase C18 column (4.6 mm×250 mm, 5 μm, Hypersil-ODS2, China). It was applicable to samples with a concentration between 2.6–209 mg/L and samples diluted to fall into this concentration range. The mobile phase was composed of acetonitrile-MKP solution (20 mmol/L) with *V*/*V* ratio of 55:45. The flow rate was 1 mL/min. The detector wavelength (UV) used was 270 nm. The temperature of the column was adjusted to 30°C. The injection dose was 10 μL. The entire running time was 10 mins. Under these chromatographic conditions, the standard linear regression equation was A = 55403C–36823 (A: peak area, C: concentration of reserpine, mg/L), R^2^ = 0.9999. Each determination was repeated three times and the results obtained were their average value.

The pH value of the solution was measured by the LEI-CI pH meter (pHS-25, INESA, China) with a combination electrode. Fourier Transform Infrared Spectrometer (FTIR-8400S, SHIMADZU, Japan) analysis was used to identify different chemical functional groups present on the SACEF before and after reserpine adsorption. The analysis was carried out using KBr and the spectral range varied from 4000 to 400 cm^−1^. The surface morphology of the SACEF before and after reserpine adsorption was analyzed by scanning electron microscopy (SEM S-3400N, HITACHI, Japan) at an accelerating voltage of 20 kV. The SACEF for FTIR and SEM was prepared in 500mL conical flask filled with 200mL reserpine solution of 10.0210 g/L and 0.5000g preprocessed SACEF. The pH value of the solution was adjusted to 5 by 0.1mol/L HCl. The flask was placed in the Magnetic Heated Stirrer (DF-101S, Yuhua, China) at 100 rpm and 30°C. A small amount of the SACEF was taken out from the conical flask at different contact time (1, 5, 10, 90min), respectively. Then the above fiber was washed by deionized water until the pH value of washing solutions reached 7. At last the fiber was dried in a vacuum oven at 60°C until the weight reached a constant. The adsorbed and dried SACEF was divided into two parts: one part for SEM and the other part for FTIR.

### 2.4 Batch adsorption experiments [24]

To study the effects of parameters such as adsorbent dosage, solution pH and temperature for reserpine adsorption onto the SACEF, the batch adsorption experiments were carried out in a series of 250 mL conical flasks with ground glass stoppers. Each flask was filled with 100 mL known initial concentration of reserpine solution and accurately weighed quantity of pretreated SACEF. The flasks were placed in the Magnetic Heated Stirrer at a constant speed (100 rpm) and a desired temperature until the adsorption equilibrium was reached. Samples were taken at the equilibrium time in above experiments. In order to study the effects of contact time and initial concentration on reserpine adsorption, samples were taken at different intervals until the adsorption reached equilibrium. The reserpine concentrations in the samples at equilibrium time (*c*_e_) and at any time (*c*_t_) were determined by HPLC. All experiments were performed in triplicates and the results obtained were their average values. The amounts of reserpine adsorbed on the SACEF at equilibrium (*q*_e_) and at any time (*q*_t_) were calculated by Eqs (1) and (2), and the percentage removal of reserpine was calculated by (Eq 3).

$$q_{\text{e}}=(c_{\text{0}}-c_{\text{e}})V/m$$ (1)

$$q_{\text{t}}=(c_{\text{0}}-c_{\text{t}})V/m$$ (2)

$$\text{Removal }\%=\frac{c_{0}-c_{e}}{c_{0}}\times \text{ 100\%}$$ (3)

Where *q*_e_ and *q*_t_ are the adsorption capacities of reserpine (mg/g) at equilibrium and at time *t* (min), respectively; *V* is the volume of solution treated, L; *c*_0_ is the initial reserpine concentration, mg/L; *c*_e_ and *c*_t_ are the reserpine concentrations at equilibrium and at time *t*, respectively, mg/L; *m* is the mass of the SACEF, g. Removal% is the percentage removal of reserpine at equilibrium.

## Results and Discussion

### 3.1 Effect of SACEF dosage on the adsorption

The number of available sites and exchanging ions for the adsorption depends on the amount of adsorbent in the adsorption process. So the SACEF dosage determines the adsorption capacity of adsorbent for a given initial concentration of reserpine solution. Different amounts of SACEF (varying from 0.02 to 0.20 g) were respectively added into initial concentration of 100 mg/L reserpine solution at 303K and at pH = 5 until the equilibrium was reached.

**Fig 2:**
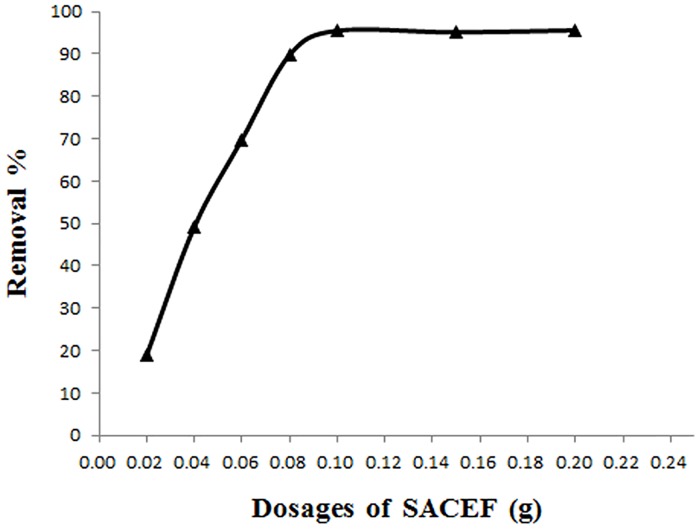
The effect of SACEF dosage on the percentage removal of reserpine. (experimental conditions: *c*_0_ = 100 mg/L, *T* = 303 K, pH = 5 and contact time = 90min).

The effect of SACEF dosage on the adsorption was shown as Fig 2, The percentage removal of reserpine increased with the increase of SACEF dosage from 0.02 to 0.10 g because more SACEF dosage could provide more surface functional groups and adsorption sites for reserpine, which increased the adsorption capacity. The percentage removal could reach 95.5% when the SACEF dosage was 0.10 g, but the increase in removal efficiency was negligible when the SACEF dosage was more than 0.10 g and this might be due to reduction in concentration gradient. So 0.10 g was selected as the SACEF dosage for the following experiments.

### 3.2 Effect of solution pH on the adsorption

The solution pH is an important controlling parameter in the adsorption process. The pH value affects the surface charge of the adsorbent and the degree of adsorbate ionization/dissociation during the adsorption process [25]. In the pH studies, the initial pH values of reserpine solution were adjusted to 1, 3, 5, 7, 9, 11 and 13 by 0.1 mol/L HCl and 0.1 mol/L NaOH, respectively. The initial reserpine concentration was 100 mg/L. The contact temperature was 303 K and the contact time was 90 mins.

**Fig 3:**
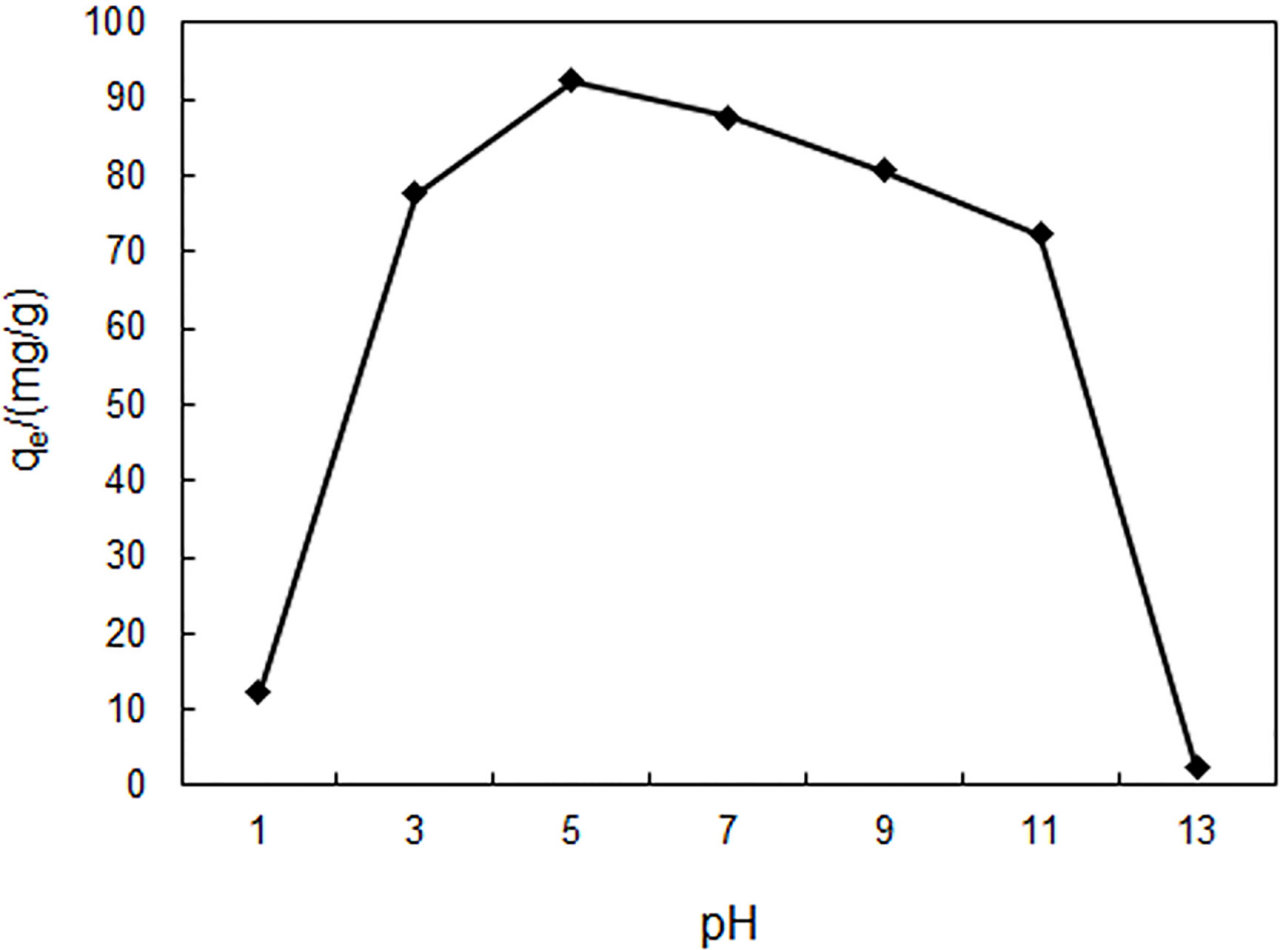
The effect of pH on equilibrium absorption capacity. (experimental conditions: *c*_0_ = 100 mg/L, *T* = 303 K, contact time = 90min and absorbent dosage = 0.10g).

Fig 3 presented the effect of pH on the adsorption of reserpine onto the SACEF. The adsorption capacity of the SACEF for reserpine depended on pH value strongly, and the maximum adsorption capacity (95.5 mg/g) was obtained at pH = 5. The adsorption capacity decreased slowly with the decrease of pH value from 5 to 3 and the increase from 5 to 11, but decreased sharply blow 3 and above 11. The pH dependency of adsorption capacity could be explained by the ionization of reserpine and the dissociation of functional group (-SO_3_H) on the SACEF. Because ionization constant of reserpine is pKa = 6.07 [26], reserpine mainly exists in its cation form in solution when pH is less than 6. This form of reserpine could easily exchange with functional group (-SO_3_H) on the SACEF, causing the increase of the adsorption capacity. But when the pH was less than 5, the excess hydrogen ions (H^+^) competed with the reserpine cation for the active sites on the surface of SACEF. The decrease of available sites for reserpine led to lower adsorption capacity. When pH was more than 6, the existence form of reserpine in solution would change from ionic state to molecular state and the degree of protonation of-SO_3_H on the SACEF would reduce because of the decrease of H^+^ concentration, which reduced the adsorption capacity of reserpine onto the SACEF.

These pH effect studies showed that the subacid condition (pH = 5) was better for the adsorption of reserpine onto the SACEF, which was distinct from reserpine adsorption onto ion exchange resin in strongly acidic condition (pH = 1) [8, 10]. Subacid solution could protect equipment and the operators from damage caused by strong acid. So the adsorption of reserpine onto the SACEF could be more safe and more maneuverable than onto the ion exchange resin. Accordingly, the initial pH of the solution was fixed at 5 for all the following experiments.

### 3.3 Effect of temperature on the adsorption

**Fig 4:**
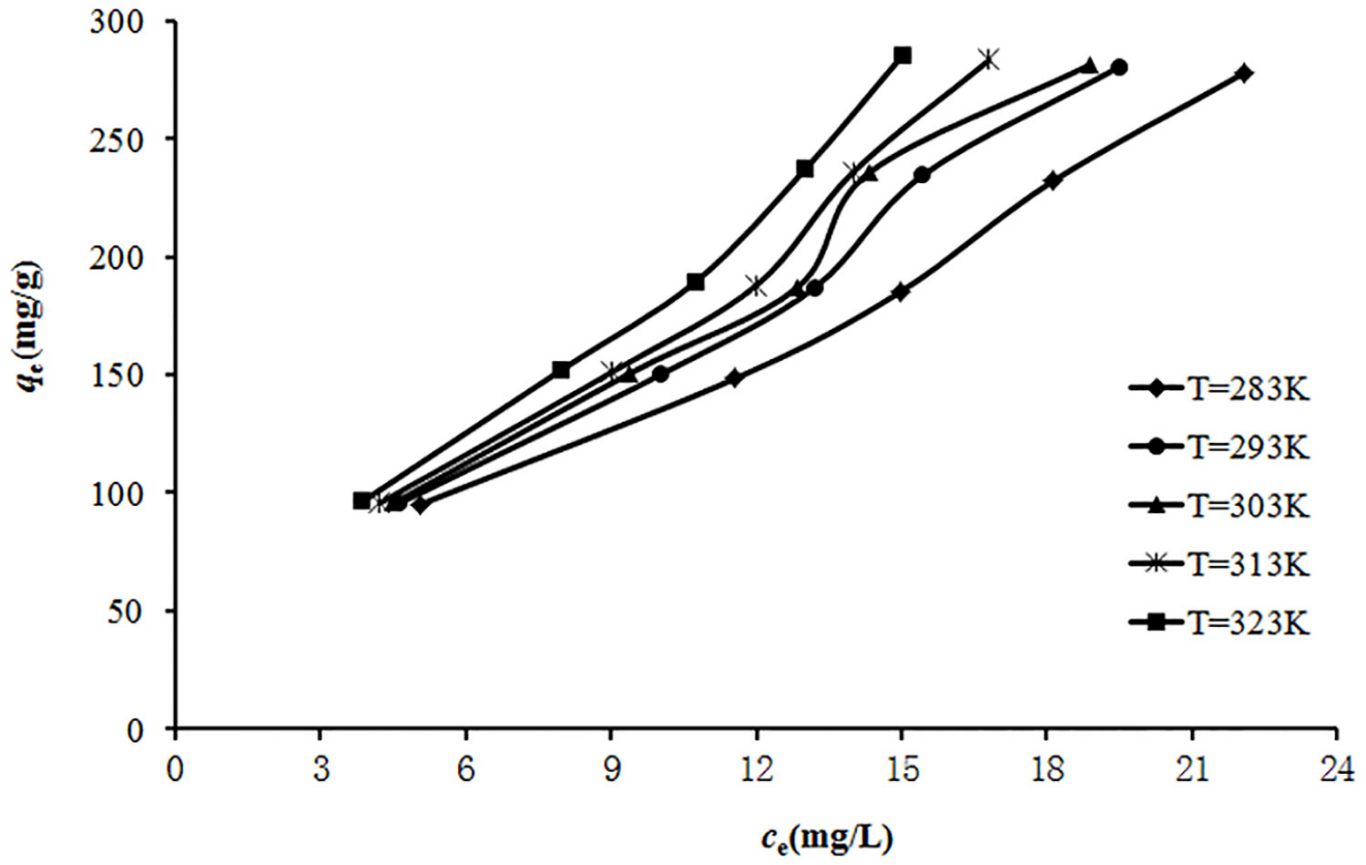
Adsorption isotherms of reserpine onto the SACEF at 283, 293, 303, 313 and 323 K. (experimental conditions: pH = 5, *c*_0_ = 100~300 mg/L and absorbent dosage = 0.10g).

The temperature is an indicator for the adsorption nature whether it is an exothermic or endothermic process. The adsorption equilibrium isotherm reflects the effect of temperature. The effects of the temperature on the adsorption of reserpine onto the SACEF in terms of adsorption isotherms at 283, 293, 303, 313 and 323K were shown as Fig 4, respectively.

As seen from Fig 4, equilibrium absorption capacity (*q*_e_) increased with the increase of equilibrium reserpine concentration (*c*_e_) at the initial concentration range between 100 mg/L and 300 mg/L. This was a result of the increase in the driving force from the concentration gradient. Under the same conditions, the active sites on the SACEF were surrounded by much more reserpine cations with the increase of *c*_e_, and the process of adsorption would carry out sufficiently. Therefore, the values of *q*_e_ increased with the increase of *c*_e_. Furthermore, when other conditions were invariant, the equilibrium absorption capacity increased with the increase of temperature, implying that the adsorption was an endothermic process. This might be due to the fact that the mobility of reserpine was better and the number of active sites on the SACEF became more at a higher temperature. The thermodynamics parameters calculations (enthalpy change, entropy change, and free energy change) in the following section would quantify the temperature effect.

### 3.4 Effect of contact time and initial concentration on the adsorption

Equilibrium time is one of the most important parameters in the design of economical separation system. To investigate the kinetics of adsorption, five different initial concentrations of reserpine were chosen, including 100, 160, 200, 250 and 300 mg/L. The experiments were carried out at 303K and at pH = 5.

**Fig 5:**
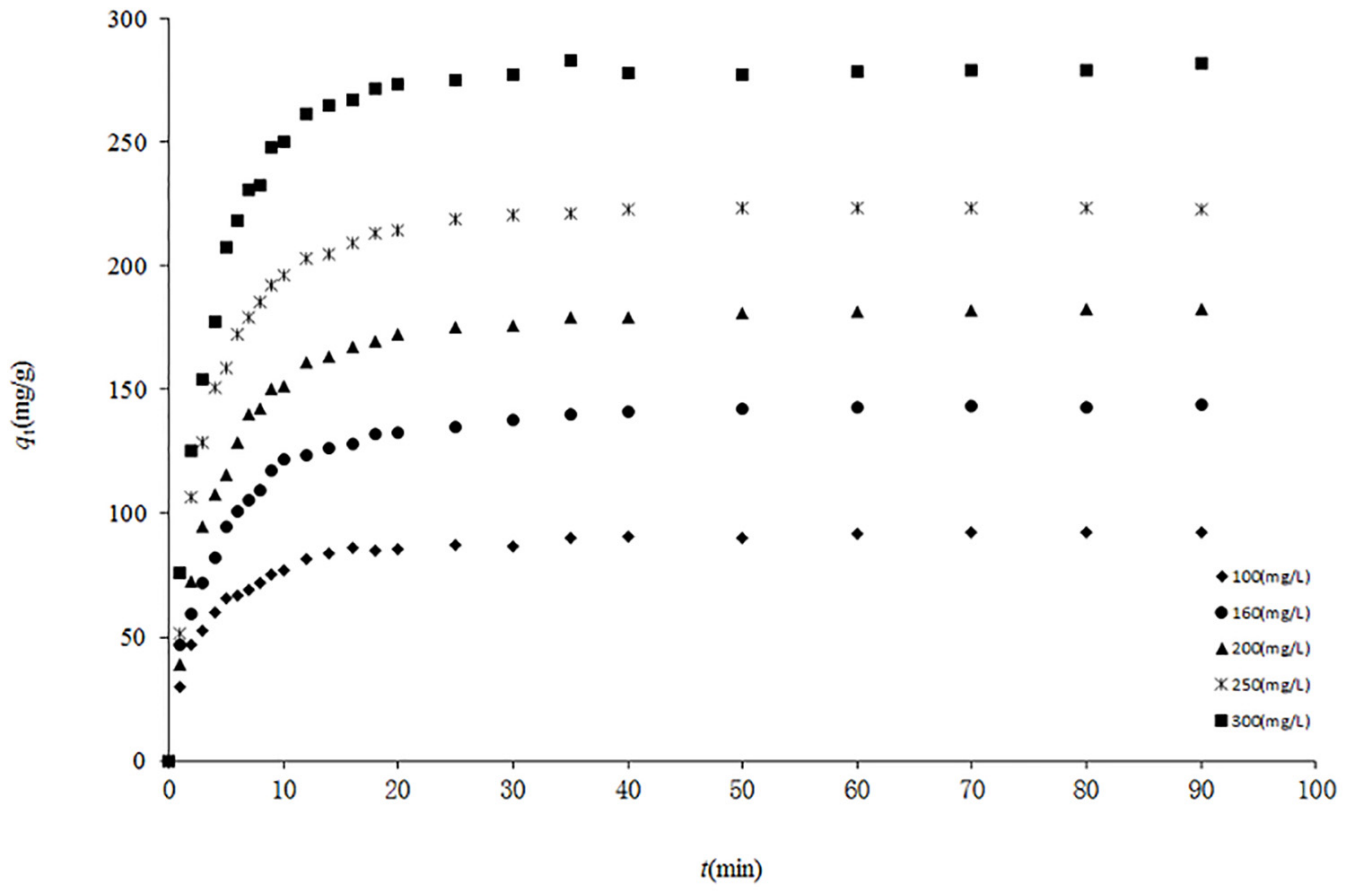
The curves of relations between *q*_*t*_ and *t* at different initial concentrations. (experimental conditions: *T* = 303 K, pH = 5 and absorbent dosage = 0.10g).

The relation between the reserpine adsorption capacity (*q*_t_) and contact time (*t*) for different initial reserpine concentrations was shown in Fig 5. The initial reserpine concentration played an important role in the adsorption of reserpine onto the SACEF. The adsorption capacity of reserpine increased evidently from 95.50 to 281.13 mg/g with the increase of initial reserpine concentrations from 100 to 300 mg/L. The reason for the increase of adsorption capacity with the increase of the initial reserpine concentration perhaps was attributed to the increase in the driving force of concentration gradient with the initial concentration increasing [24]. And also as shown in Fig 5, the adsorption capacity increased with the increase of contact time. The adsorption of reserpine onto the SACEF was fast before 10 min, achieving over 83% of equilibrium adsorption capacity, and the equilibrium was nearly reached after 20 min. This process of adsorption showed that there might be a large number of vacant surface sites available for adsorption during the initial stage, then, after 10 min the remaining vacant surface sites were more and more difficult to be occupied because of the repulsive forces between the reserpine ions on the SACEF and the bulk phase [27]. The adsorption reached equilibrium within 20 mins, implying that the SACEF displayed an excellent kinetic property for adsorbing reserpine.

### 3.5 Kinetic studies

Adsorption kinetics describes the rate of adsorbate uptake governing the contact time of adsorption, which is an important characteristic defining the efficiency of adsorption [28]. It is controlled by the adsorption mechanism and the rate-limiting step of the process [29]. In order to investigate the adsorption kinetics, the adsorption kinetics data at the temperature of 303 K (shown as Fig 5) were chosen. The adsorption mechanism was evaluated by the pseudo-first-order and pseudo-second-order models, while the rate-limiting step was evaluated by the Weber and Morris intra-particle diffusion model.

The pseudo-first-order model is one of the most widely used adsorption model for the adsorption of adsorbate from a liquid solution. The liner form of pseudo-first-order model equation [30] is as following:

$$\text{ln}(q_{\text{e}}-q_{\text{t}})=\text{ln}q_{e}-k_{1}t$$ (4)

**Fig 6:**
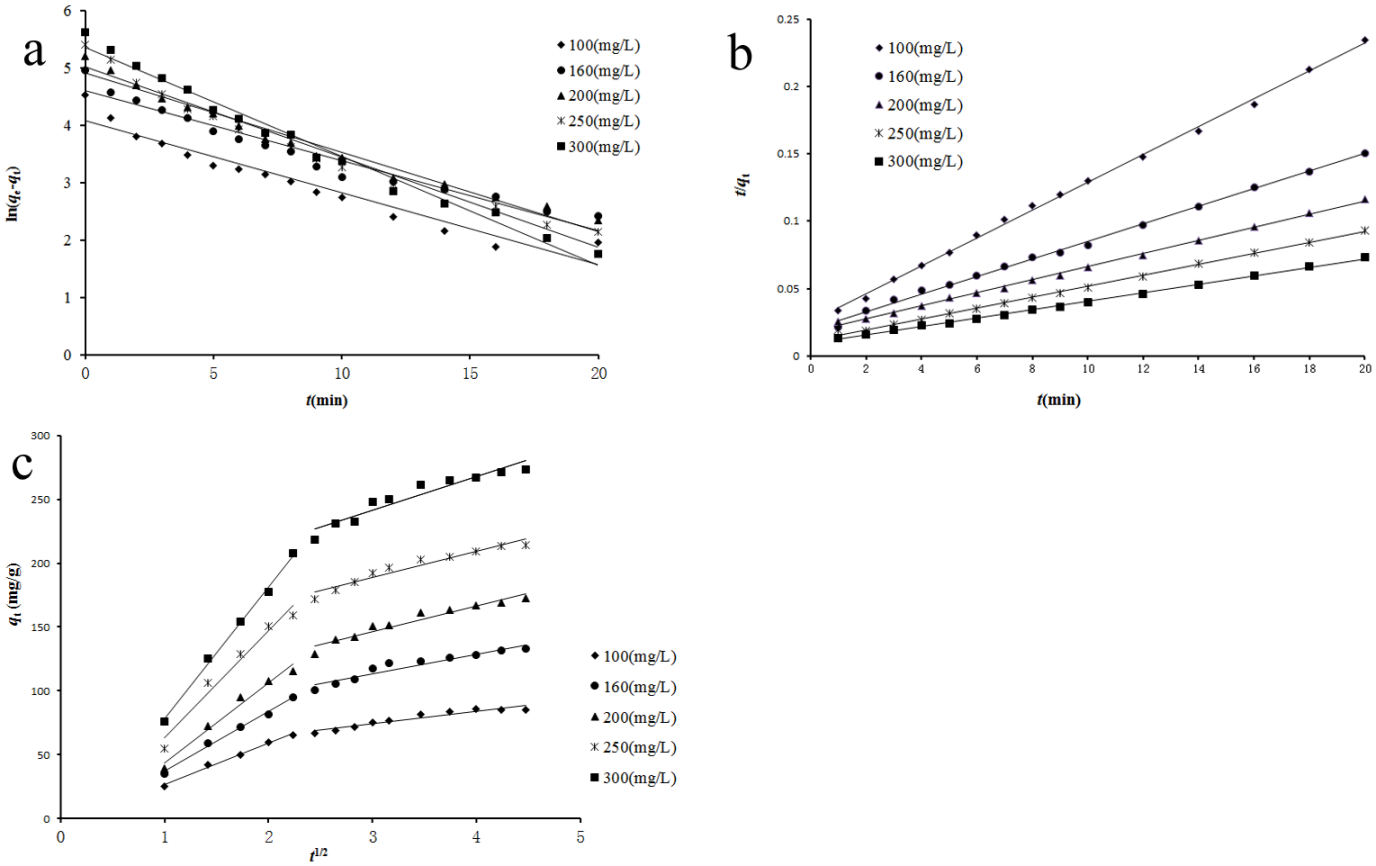
Regressions of kinetics plots at different initial reserpine concentration. (experimental conditions: *T* = 303 K, pH = 5, *c*_0_ = 100~300 mg/L and absorbent dosage = 0.10g). (a) pseudo-first-order; (b) pseudo-second-order; (c) intra-particle diffusion.

Where *q*_e_ and *q*_t_ are the adsorption capacities of reserpine (mg/g) at equilibrium and at time *t*, respectively, and *k*_1_ is the rate constant of pseudo-first-order adsorption (min^-1^). *k*_1_ and *q*_e_ can be calculated from the linear plots of ln(*q*_e_-*q*_t_) versus *t* (Fig 6a). The results at different initial reserpine concentrations were listed in Table 1.

**Table 1:** Kinetics parameters of pseudo-first-order, pseudo-second-order and Weber and Morris intra-particle diffusion of reserpine adsorption onto the SACEF.

initial concentration *c*_0_ (mg/L)	Pseudo-first-order				Pseudo-second-order				*q*_e(exp)_ (mg/g)	Weber and Morris intra-particle diffusion model for the second stage		
	*k*_*1*_ (min^-1^)	*q*_e(cal)_ (mg/g)	*R* ^2^	%Error	*k*_*2*_×10^−3^ (g/mg·min)	*q*_e(cal)_ (mg/g)	*R* ^2^	%Error		*R* ^2^	*k*_*i*_ (mg/(g·min^0.5^)	*I* (mg/g)
100	0.126	59.61	0.939	5.694%	4.2	97.09	0.998	2.392%	95.50	0.916	9.82	44.73
160	0.122	100.31	0.952	3.855%	2.1	153.85	0.997	3.468%	150.61	0.932	17.52	56.35
200	0.138	136.67	0.971	3.354%	1.6	188.68	0.998	2.175%	187.15	0.939	20.14	86.02
250	0.158	153.13	0.967	4.231%	1.6	232.56	0.997	2.620%	235.67	0.942	20.38	127.69
300	0.190	212.90	0.984	3.799%	1.3	285.71	0.998	2.089%	281.13	0.911	26.35	162.27

The pseudo-second-order linear equation [31] can be expressed as following:

$$\frac{t}{q_{t}}=\frac{1}{k_{2}q_{e}^{2}}+\frac{t}{q_{e}}$$ (5)

Where *k*_2_ is the rate constant of pseudo-second-order adsorption (g/ (mg·min)), *k*_2_ and *q*_e_ can be determined from the slop and intercept of the plot of *t*/*q*_t_ versus *t* (Fig 6b). The results were listed in Table 1.

In order to evaluate the fitness of the kinetic models to the experimental kinetic data, the average relative error function (%Error) was used to measure the kinetic constants and compare the kinetic models. The average relative error is calculated as following [32]:

$$\%\text{Error}=\frac{1}{h}\sum_{i=1}^{h}\frac{\left(q_{\text{exp},i}-q_{calc,i}\right)}{q_{\text{exp},i}}$$ (6)

Where the subscripts ‘‘exp” and ‘‘calc” show the experimental and calculated values and *h* is the number of measurements. Smaller %Error value indicates a better fitness. The results were listed in Table 1.

It was obvious from Fig 6a and 6b, Table 1 that the pseudo-second-order model fitted the experimental data better than the pseudo-first-order model for the entire adsorption, with better correlation coefficients (R^2^>0.99) and lower average relative errors (%Error<3.5%). The values of *q*_e(cal)_ calculated from the pseudo-second-order model were closer to *q*_e(exp)_ obtained from the experiments than those calculated from the pseudo-first-order model, further confirming the feasibility of the pseudo-second-order model. Furthermore, the values of the rate constant *k*_2_ were found to decrease with the increase of the initial reserpine concentrations in Table 1. The reason for this behavior perhaps was attributed to the less competition for the surface active sites at lower concentrations, but the higher competition at higher concentrations [25].

Since neither the pseudo-first-order model nor the pseudo-second-order model could identify the diffusion mechanism and rate-limiting step, the kinetic data were analyzed by Weber and Morris intra-particle diffusion model to elucidate the diffusion mechanism and rate-limiting step. Weber and Morris intra-particle diffusion model assumes that the adsorption can occur through three consecutive steps including film diffusion, intra-particle diffusion and sorption. Since sorption is a fast and non-limiting step in the adsorption process, the adsorption rate can be limited by film diffusion or/and intra-particle diffusion [25]. The Weber and Morris intra-particle diffusion model [33] is expressed as following:

$$q_{t}=k_{t}t^{1/2}+I$$ (7)

Where *k*_i_ is the rate constant of intra-particle diffusion. *I* is a constant that gives idea about the thickness of the boundary layer (mg/g). The larger the value of *I*, the greater the boundary layer effect is [34]. The values of *k*_i_ and *I* can be determined from the slope and intercept of the linear plot of *q*_t_ versus *t* ^1/2^, respectively. If the plot of *q*_t_ versus *t* ^1/2^ gives a straight line and passes through the origin, the adsorption process is controlled only by the intra-particle diffusion. But if the plot shows multi-linear characteristic or does not pass through the origin, the adsorption process is controlled by two or more diffusion mechanisms [35]. In this study, the plot of *q*_t_ versus *t* ^1/2^ was shown in Fig 6c, and the values of *k*_i_ and *I* were listed in Table 1.

As seen from Fig 6c, the plots of different initial reserpine concentrations could be divided into two stages (the first stage and the second stage). This could be attributed to the fact that in the first stage the adsorption was due to boundary layer diffusion effect whereas in the second stage it was due to the intra-particle diffusion effect [25]. The regressions for the two stages were linear (*R* ^2^>0.91), but the plots did not pass through the origin, suggesting that the adsorption involved intra-particle diffusion, but that was not the only rate-limiting step in the adsorption process of reserpine onto the SACEF. The adsorption mechanism could possibly be controlled by multi-diffusion steps, including film diffusion step and intra-particle diffusion step. The values of the intra-particle diffusion rate *k*_i_ and *I* were obtained from the slope and intercept of the second stage, respectively (shown in Table 1). The results showed that the values of *k*_i_ and *I* increased with the increase of the initial concentrations (100-300mg/L), demonstrating that the increase of the initial concentration could promote the adsorption process.

### 3.6 Activation energy

The activation energy is an important parameter to help to elucidate the mechanism occurring for the adsorption, whether the sorption is a physisorption or chemisorption process [36]. The Arrhenius equation is used to correlate the activation energy and rate constant of kinetic model at different temperatures. The Arrhenius equation linear form is as following:

$$\text{ln}k=-\frac{Ea}{RT}+\text{ln}A$$ (8)

Where *k* is *k*_2_ of the pseudo-second-order model at different temperature since the pseudo-second-order model was identified as the best kinetic model for the adsorption of reserpine onto the SACEF; *R* is the gas constant, 8.314 kJ/(mol·K); *T* is adsorption temperature, K; *Ea* is activation energy, kJ/mol, and *A* is the frequency factor for a given reaction.

From the kinetic data obtained at different temperatures, the rate constants (*k*_2_) of pseudo-second-order model were 0.0020, 0.0020, 0.0021, 0.0023 and 0.0024 at 283, 293, 303, 313 and 323 K, respectively. Arrhenius equation was given as ln*k* = -523.05/*T*-4.3987 (*R* ^2^ = 0.9537) by the plot of ln*k* versus 1/*T* and *Ea* was calculated to be 4.35 kJ/mol from this equation. For the value of *Ea* less than 40 kJ/mol, the adsorption process of reserpine onto the SACEF was a rapid physisorption process [36]. This was one of the important reasons why the adsorption of reserpine onto the SACEF could reach the equilibrium in such a short period of time (20 mins).

### 3.7 Adsorption isotherm

Adsorption isotherm describes how the adsorbate molecules distribute between the liquid and the solid phases when the adsorption process reaches an equilibrium state [37]. The analysis of the adsorption isotherms data by fitting them into different isotherm models is an important step to find the suitable model that can be used for design process. It was found that adsorption equilibrium time of reserpine onto the SACEF was 20 mins. The adsorption isotherms of reserpine on the SACEF at 283, 293, 303, 313 and 323 K were shown in Fig 4, respectively. The Langmuir, Freundlich, Dubinin-Radushkevich (D-R) and Temkin isotherm models were used to describe the equilibrium adsorption, respectively.

The Langmuir isotherm model describes monolayer adsorption onto homogeneous surface with no interaction between adjacent adsorbed molecules. The linear form of Langmuir isotherm model is as following [38]:

$$\frac{1}{q_{e}}=\frac{1}{q_{m}k_{L}c_{e}}+\frac{1}{q_{m}}$$ (9)

**Fig 7:**
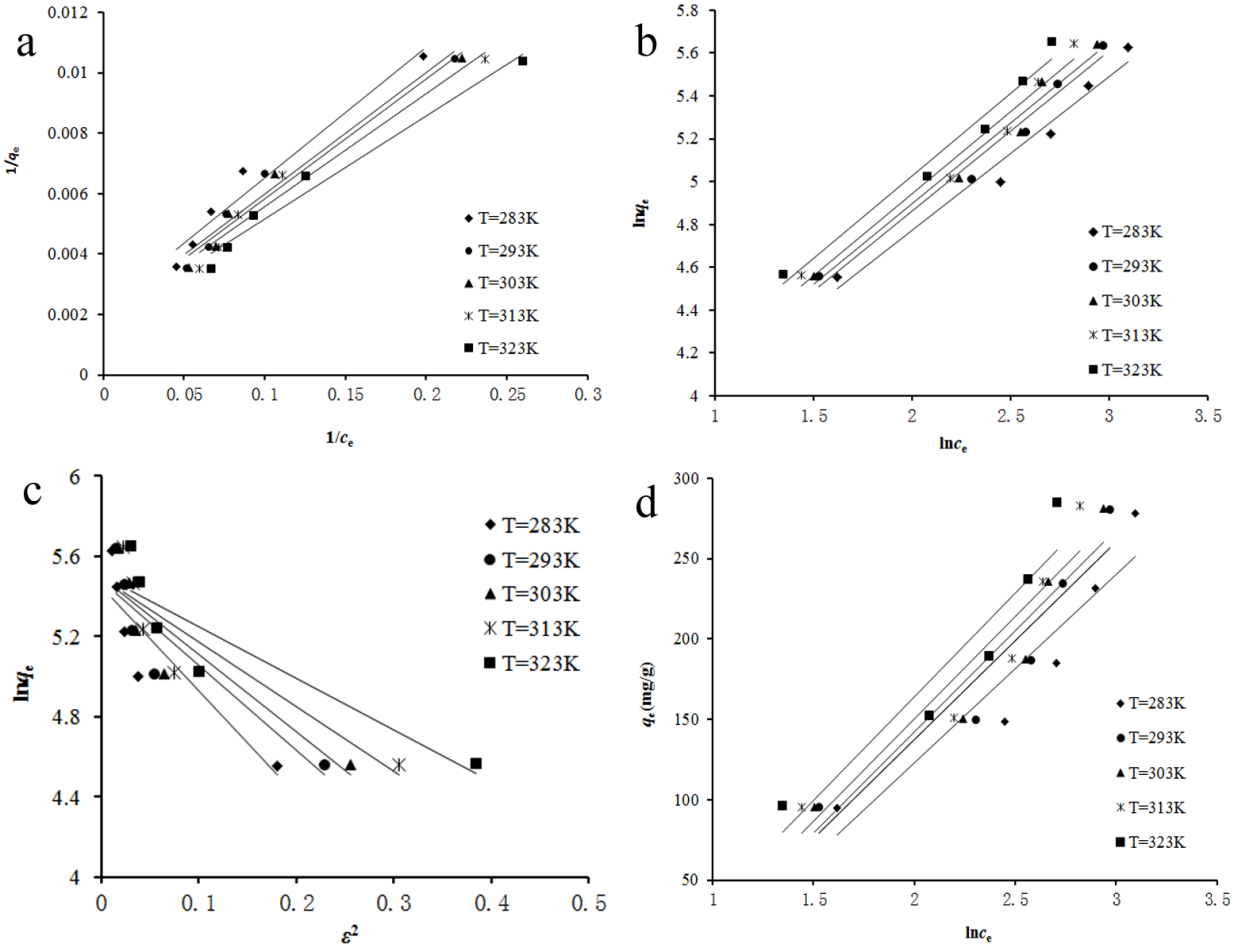
The Langmuir, Freundlich, D-R and Temkin adsorption isotherms from 283 to 323 K. (a) Langmuir isotherm;(b) Freundlich isotherm;(c) D-R isotherm;(d) Temkin isotherm.

Where *q*_e_ is the equilibrium adsorption capacity, mg/g; *c*_e_ is the equilibrium concentration of reserpine in solution, mg/L; *k*_L_ is the Langmuir constant, L/mg, which is related to the affinity of binding sites; and *q*_m_ is the theoretical saturation capacity of the monolayer, mg/g. The values of *q*_m_ and *k*_L_ were derived from the intercept and slope of the linear plot of 1/*q*_e_ versus 1/*c*_e_ shown in Fig 7a and listed in Table 2.

**Table 2:** Parameters of Langmuir, Freundlich, D-R and Temkin isotherm model equations.

Isotherms	Parameters	283K	293K	303K	313K	323K
Langmuir	*k*_*L*_(L/mg)	0.048	0.048	0.048	0.049	0.050
	*q*_*m*_(mg/g)	476.19	500.00	526.32	555.56	588.24
	*R*_*L*_	0.064~0.171	0.063~0.167	0.065~0.173	0.065~0.172	0.064~0.171
	*R* ^2^	0.961	0.969	0.973	0.968	0.974
	*%Error*	8.015%	7.760%	7.613%	7.738%	7.036%
Freundlich	*k*_*F*_/(mg/g)(L/mg)^1/n^	28.23	29.28	29.52	30.09	32.44
	1/*n*	0.717	0.744	0.755	0.769	0.773
	*R* ^2^	0.970	0.975	0.975	0.971	0.976
	*%Error*	6.140%	5.789%	5.708%	6.180%	5.227%
D~R	*k*_*D*_/1/(mg·kJ) ^2^	5.21	4.21	3.84	3.21	2.58
	*q*_*m*_(mg/g)	232.73	238.96	242.35	242.91	246.34
	*R* ^2^	0.802	0.818	0.829	0.820	0.832
	*%Error*	14.599%	14.275%	14.080%	14.201%	13.684%
Temkin	*k*_*T*_ (L/mg)	0.39	0.41	0.42	0.44	0.48
	*B*	117.19	122.97	125.47	127.44	128.98
	*R* ^2^	0.894	0.905	0.911	0.893	0.900
	*%Error*	11.614%	11.302%	11.100%	11.607%	10.746%

Furthermore, another important parameter, *R*_L_, called the separation factor or equilibrium parameter is used to predict whether an adsorption system is favorable or unfavorable. And *R*_L_ is determined from the relation as following [39]:

$$R_{L}=\frac{1}{1+k_{L}c_{0}}$$ (10)

The value of *R*_L_ indicates the type of the isotherm to be either unfavorable (*R*_L_ >1), linear (*R*_L_ = 1), favorable (0<*R*_L_ <1) or irreversible (*R*_L_ = 0) [29]. The values of *R*_L_ for reserpine adsorption onto the SACEF were shown in Table 2. From Table 2, we could know that the values of *R*_L_ were less than 1 and greater than 0, indicating a favorable adsorption of reserpine onto the SACEF.

The Freundlich isotherm model is an empirical relationship applied to the adsorption on heterogeneous surfaces with the interaction between adsorbed molecules. And it suggests that sorption energy exponentially decreases on completion of the sorption centers of an adsorbent [40]. The linear form of Freundlich isotherm model is as following:

$$\text{ln}q_{e}=\text{ln}k_{F}+\frac{1}{n}\text{ln}c_{e}$$ (11)

Where *k*_F_ (mg/g(L/mg)^1/n^) and *n* are the Freundlich constants. The value of 1/*n* indicates the type of isotherm. When 1/*n*>1, the adsorption is unfavorable; when 1/*n* = 1, the adsorption is irreversible; and when 0<1/*n*<1, the adsorption is favorable [25]. Both *k*_F_ and 1/*n* could be obtained from the intercept and slope of the linear plot of ln*q*_e_ versus ln*c*_e_ shown in Fig 7b and the values were listed in Table 2. As shown in Table 2, the values of 1/*n* were less than 1, indicating that the adsorption of reserpine onto the SACEF was a favorable process.

The D-R isotherm model describes the adsorption similar to the Langmuir type, but it does not assume a homogeneous surface or constant adsorption potential. The linear form of D-R isotherm model can be described as following [41]:

$$\text{ln}q_{e}=\text{ln}q_{m}-k_{D}\varepsilon^{2}$$ (12)

$$\varepsilon =RT\text{ln}(1+\frac{1}{c_{e}})$$ (13)

Where *q*_m_ is the theoretical saturation capacity, mg/g; *k*_D_ is the constant related to adsorption energy, mg^2^/kJ^2^; *R* is the gas constant, 8.314 kJ/ (mol·K); *T* is adsorption temperature, K; *ε* is Polanyi potential. The linear plots of ln*q*_e_ versus *ε* ^2^ were shown in Fig 7c and the values of the constants were listed in Table 2.

The Temkin isotherm model describes the behavior of adsorption on a heterogeneous surface. And it assumes that the heat of adsorption of all the molecules in the layer decreases linearly with coverage due to adsorbent-adsorbate. The linear form of Temkin isotherm model is as following [42]:

$$q_{e}=B\text{ln}k_{T}+B\text{ln}c_{e}$$ (14)

Where *B* is a constant related to adsorption heat, and *k*_T_ is the equilibrium binding constant corresponding to maximum binding energy, L/mg. The linear plots of *q*_e_ versus ln*c*_e_ were shown in Fig 7d and the values of the constants were listed in Table 2.

In order to evaluate the fitness of the isotherm to the experimental equilibrium data, the average relative error function shown as (Eq 6) was also used to compare the isotherm models, and the results were also listed in Table 2.

As shown in Fig 7 and Table 2, the correlation coefficients (*R* ^2^) were larger than 0.96 and the average relative errors (%Errors) were smaller than 8% for both the Langmuir and the Freundlich models, suggesting that the two isotherm models were both suitable to predict the adsorption of reserpine onto the SACEF. Similar results had been obtained for the adsorption of salvianolic acid B [21], baicalin [17], catechin [22] onto ion exchange fiber. However, compared with Langmuir isotherm model, Freundlich isotherm model might be relatively more suitable to predict the adsorption of reserpine onto the SACEF with better correlation coefficients (*R* ^2^>0.97) and lower average relative errors (%Errors<6.2%). This result implied that the present adsorption system was surface energy heterogeneity, which was in accord with the result of the thermodynamic parameter calculation of Δ*H* (section 3.8).

Furthermore, the values of *q*_*m*_ in Langmuir isotherm models and 1/*n* in Freundlich isotherm models increased with the increase of the temperature, suggesting that the adsorption of reserpine onto the SACEF was endothermic and could be promoted by increasing temperature. This result would be further confirmed by the thermodynamic parameter calculation (section 3.8).

### 3.8 Thermodynamic parameters

Proper assessment of thermodynamics can provide in-depth information regarding the inherent energy and structural changes of adsorbent after adsorption and also provide the mechanism involved in adsorption process [43]. The thermodynamics parameters studies include enthalpy change (Δ*H*), free energy change (Δ*G*) and entropy change (Δ*S*). They can be calculated from the following equations [25][44].

$$K_{d}=\frac{q_{e}}{c_{e}}$$ (15)

$$\text{ln}K_{d}=-\frac{\Delta H}{RT}+\frac{\Delta S}{R}$$ (16)

ΔG=ΔH−TΔS (17)

Where *K*_*d*_ is the distribution coefficient, L/g; *q*_e_ is the equilibrium adsorption capacity, mg/g; *c*_e_ is the equilibrium reserpine concentration of, mg/L; *R* is the gas constant, 8.314 kJ/ (molK); And *T* is the absolute temperature, K.

**Fig 8:**
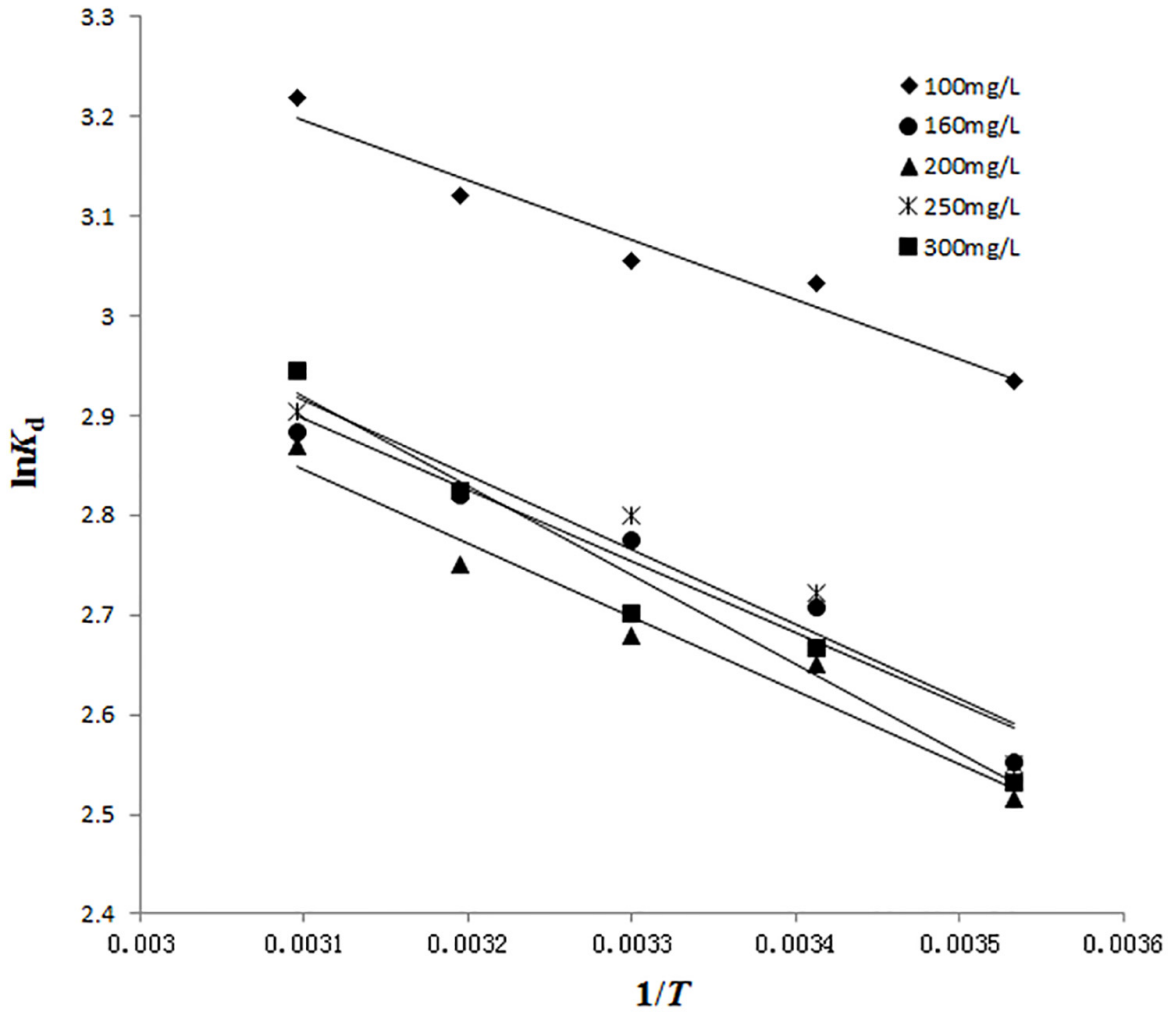
The plots of ln*K*_*d*_ versus 1/*T* at various initial concentrations. (experimental conditions: pH = 5 and *c*_0_ = 100~300 mg/L).

The plots of ln*K*_d_ versus 1/*T* at different initial concentrations were shown in Fig 8. The plots were all linear with good correlation coefficients (*R* ^2^>0.92), suggesting that Van’t Hoff equation was suitable to be applied for the calculations of enthalpy change (Δ*H*) and entropy change (Δ*S*). The values of Δ*H* and Δ*S* could be obtained from the slopes and intercepts of the plots, respectively. The results were listed in Table 3.

**Table 3:** The thermodynamics parameters of the adsorption for reserpine onto the SACEF.

*c*_*0*_ (mg/L)	Δ*H* (kJ/mol)	Δ*S* (J/(mol·K)	Δ*G* (kJ/mol)				
			283K	293K	303K	313K	323K
100	4.97	41.97	-11.87	-12.29	-12.71	-13.13	-13.55
160	5.94	42.50	-12.02	-12.45	-12.87	-13.30	-13.72
200	6.15	42.72	-12.08	-12.51	-12.94	-13.37	-13.79
250	6.21	43.50	-12.30	-12.74	-13.17	-13.61	-14.04
300	7.44	47.35	-13.39	-13.87	-14.34	-14.81	-15.29

As shown in Table 3, positive values of Δ*H* suggested the endothermic nature of the adsorption process of reserpine onto the SACEF. The adsorption process would become more favorable at a higher temperature, which was in accord with the adsorption isotherm. Similar phenomena had been observed for the adsorption of salvianolic acid B [21], baicalin [17], catechin [22] onto the ion exchange fibers. The increase of Δ*H* with the increase of initial reserpine concentration reflected the heterogeneity of surface energy of the SACEF [45]. The reason for the increase of Δ*H* might be that the process of adsorption occurred on the most active sites at the beginning of adsorption with low energy needed. With the progress of the adsorption process, the active sites decreased and further adsorption became more difficult with higher energy needed [28]. When the initial reserpine concentration range was between 100 and 300 mg/L, the values of Δ*H* were between 4.97 and 7.44 kJ/mol, which were less than 40 kJ/mol, indicating that the adsorption process of reserpine onto the SACEF was controlled by physical mechanism [29].

The values of the free energy change (Δ*G*) were negative and decreased with temperature increasing (shown as Table 3), indicating that the adsorption of reserpine onto the SACEF was spontaneous and the adsorption became more favorable at a higher temperature. In addition, the absolute values of Δ*G* were smaller than 20 kJ/mol, implying that the adsorption of reserpine onto the SACEF was a physisorption process [46].

Furthermore, the positive values of Δ*S* as shown in Table 3 indicated that the degrees of randomness increased at the solid-liquid interface during the adsorption of reserpine onto the SACEF. This could be explained from “solvent replacement” [45, 46]. The SACEF was hydrophilic because of the functional group (-SO_3_H) on the surface. Before reserpine was adsorbed on the surface of the SACEF, the existing bonds between the SACEF molecules and water molecules had to be broken and then reserpine molecules would take the place of the water molecules and form possible bonds with the SACEF. This process was called “solvent replacement”. As the molar volume of water molecule was much smaller than that of reserpine molecule, the replaced water molecules were more than the adsorbed reserpine molecules, leading to the increase of Δ*S*.

### 3.9 SEM analysis of the adsorption process of reserpine on SACEF

**Fig 9:**
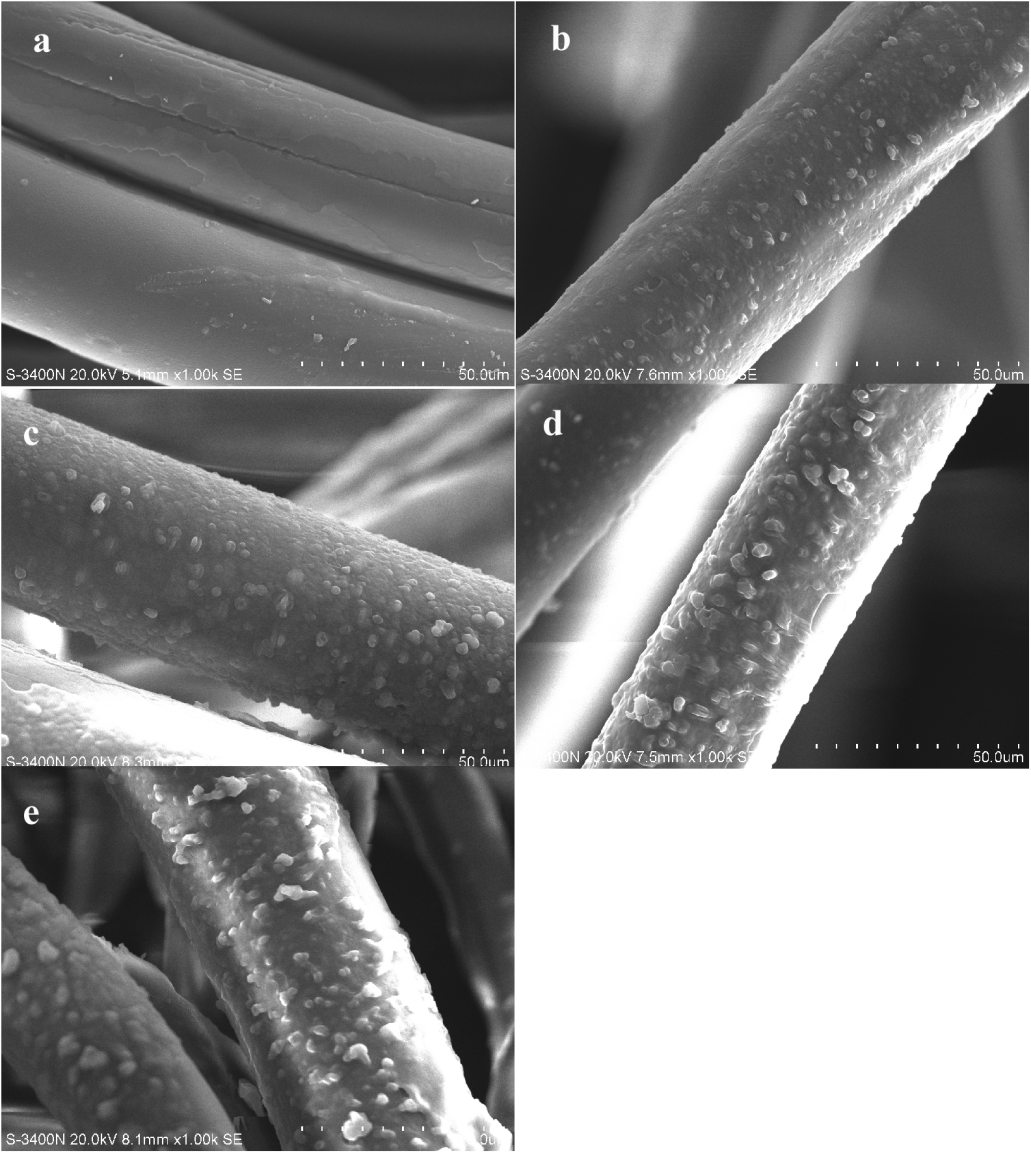
The SEM images of the SACEF adsorbing reserpine at different contact time. (a) SACEF before adsorption; (b) SACEF adsorbing reserpine after 1 min;(c) SACEF adsorbing reserpine after 5 mins;(d) SACEF adsorbing reserpine after 10 mins;(e) SACEF adsorbing reserpine after 90 mins.

The SEM images of the SACEF adsorbing reserpine at five different contact time (0, 1, 5, 10, 90min) were shown as Fig 9, respectively.

The SEM images from Fig 9a–9e had shown the dynamic process of reserpine adsorbed onto the SACEF. As shown in Fig 9a, the surface of the SACEF before adsorption appeared to be smooth or/and irregularly scarred, with nothing adhering to the surface of the SACEF. However, white scattered spots appeared on the SACEF surface after 1 minute of adsorption (as shown in Fig 9b). Then the spots on the SACEF surface became more and more with the increase of adsorption time (Fig 9c), and agglomerations appeared on the SACEF surface after 10 mins (Fig 9d). At last the SACEF surface was covered with much more and bigger agglomerations after 90 mins (Fig 9e). The component adsorbed on the surface of SACEF should be reserpine according to the preparation of SACEF for SEM and FTIR in section 2.3.

### 3.10 FTIR analysis of the SACEF adsorbing reserpine

**Fig 10:**
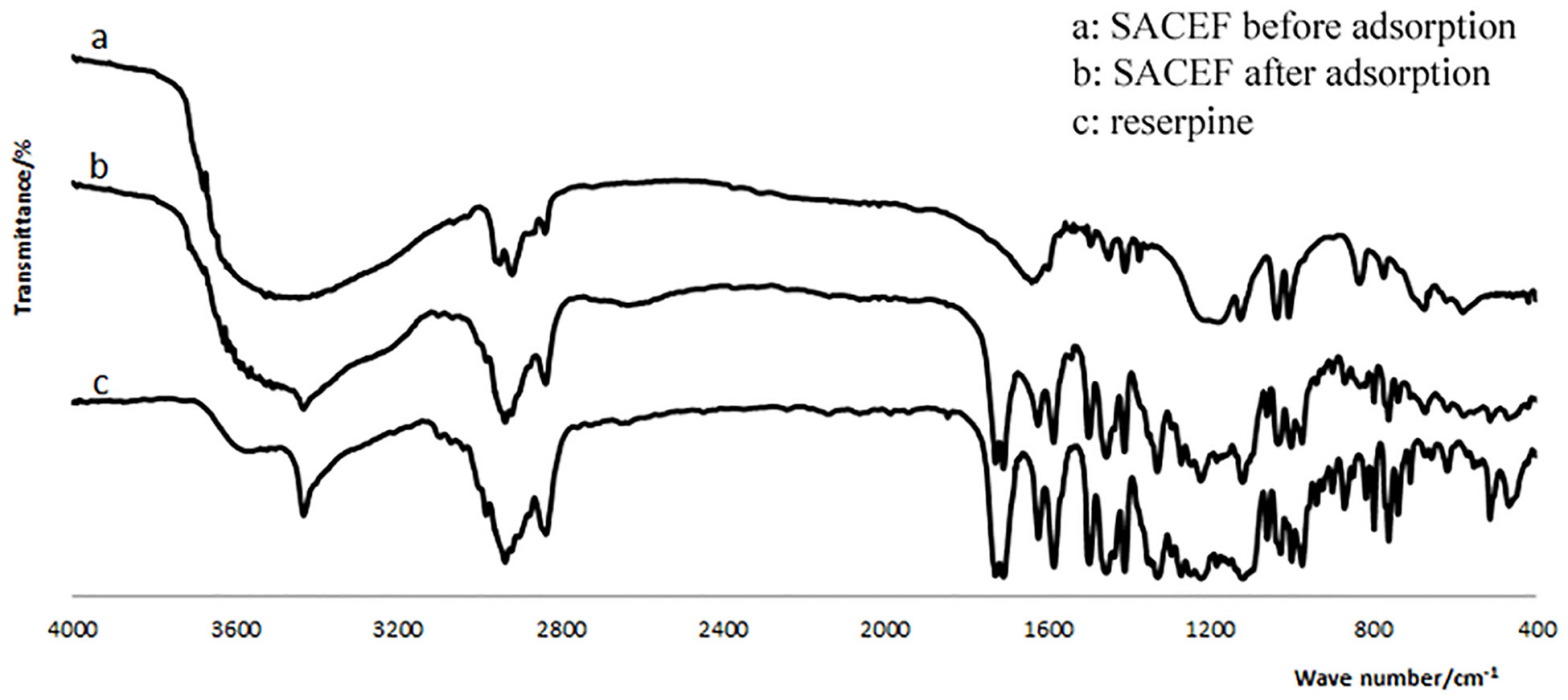
FTIR spectra of the SACEF before and after adsorption of reserpine.

In order to further verify the component adsorbed on the surface of SACEF (shown as SEM images) was reserpine, FTIR had been carried out. The FTIR spectra before and after adsorption of reserpine onto the SACEF had been shown as Fig 10. Compared with the FTIR spectra of reserpine (Fig 10c) and the SACEF before adsorption (Fig 10a), the FTIR spectrum of the SACEF after adsorption (Fig 10b) not only kept the characteristic peaks of the SACEF, but also emerged characteristic peaks of reserpine at 1700, 1730 and 3437 cm^-1^, which could be assigned to double C = O and N-H of reserpine, respectively. These results of the FTIR spectra further confirmed that the component adsorbed on the surface of SACEF was reserpine accurately.

## Conclusions

The current study has provided insights into the adsorption of reserpine onto the SACEF. The adsorption was found to be pH dependent and the optimum reserpine adsorption onto the SACEF occurred at the subacid condition (pH = 5) of reserpine solution. The SACEF had high adsorption rate for reserpine since over 83% of the equilibrium adsorption capacity was achieved during the first 10 min and the equilibrium was attained within 20 mins. The kinetics of the adsorption followed the pseudo-second-order model, and the intra-particle diffusion was not the sole rate-limiting step in the adsorption process. The activation energy *Ea* was calculated to be 4.35 kJ/mol, implying the adsorption of reserpine onto the SACEF was a rapid physisorption process. Under the studied conditions, thermodynamic calculations showed the endothermic, spontaneous and feasible nature of the adsorption and the equilibrium data could preferably be modeled with the Freundlich isotherm. Furthermore, the successful adsorption of reserpine onto the SACEF was confirmed by SEM and FTIR. The conclusion was obtained that the SACEF was an efficient adsorption material for reserpine.
